# Gut microbiota analysis in inflammatory bowel disease: Novel findings from old data

**DOI:** 10.1002/ueg2.12518

**Published:** 2023-12-29

**Authors:** Irene Marafini, Giovanni Monteleone

**Affiliations:** ^1^ Gastroenterology Unit Policlinico Universitario Tor Vergata Rome Italy; ^2^ Department of Systems Medicine University of Rome “Tor Vergata” Rome Italy

**Keywords:** Crohn's disease, IBD, microbiota, ulcerative colitis

The presence of altered gut microbiota, usually described as intestinal “dysbiosis,” has been widely documented in inflammatory bowel disease (IBD) and it is thought to influence the onset and perpetuation of gut inflammation both in Crohn's disease (CD) and ulcerative colitis (UC).^1^ Dysbiosis is characterized by the reduction of beneficial bacteria, overgrowth of potentially pathogenic bacteria, and loss of bacterial diversity.^2^ Despite the enormous amount of scientific work devoted to the study of gut microbiota in IBD, our understanding of how to modulate the flora for therapeutic purposes is still very limited. Gut microbiota analyses are influenced by technical differences, that lead to extremely variable results across studies and this may in part account for the paucity of clinical implications for the data obtained. The Microbiome Quality Control project, which is designed to comprehensively evaluate methods for measuring the human microbiome, identify sources of variation in microbiome studies, quantify their magnitudes, and assess the design and utility of different positive and negative control strategies, has evidenced that each step of the process may lead to different biases that influence the final results.^3^, ^4^ For example, microbiota analysis may significantly vary depending on sample preservation methods, type of extraction, the choice of sequences to be amplified, and the statistical methods used for bioinformatic analysis.

In this issue of the United European Gastroenterology Journal, Vestergaard and co‐workers aimed to identify, combine, and re‐analyze original 16S rRNA amplicon sequencing data from internationally available cohorts of patients with IBD and healthy individuals to determine robust microbiota signatures for IBD.^5^ The purpose of the study is that using a larger sample size and taking into account technical variations among studies could provide more reliable results. The author searched the BioProject database for original fecal 16S rRNA amplicon sequencing datasets from adult IBD patients and healthy individuals worldwide. Forty‐five datasets including 5381 stool samples from 36 different countries, harboring 1345 different genera, were considered eligible for the study. Information on disease status, sex, and age was available for 934 patients with IBD (691 CD and 243 UC) and 1584 healthy individuals. The number of tested genera was limited to 144 following the exclusion of all genera present in less than 10% of the samples. Technical and biological confounders were adjusted in the final analysis. The study confirmed reduced alpha and beta diversity in IBD patients compared to healthy controls and the presence of a different microbiota composition between IBD and healthy controls and between CD and UC, and identified 94 genera differentially abundant between patients withIBD and healthy individuals. Of these, 38 genera in CD and 28 genera in UC represented novel associations, which were not identified by analyses of the included individual cohorts or previous meta analyses of these cohorts. Some of these newly identified genera have possible functional roles described in the literature that strengthen these findings and lay the foundation for further investigations. For example, in CD, there was a relative abundance of the genera *Atopobium*, which has been associated with the severity of gut inflammation in mouse models of IBD and with mitochondrial dysfunction documented in children with IBD.^6^ Moreover, a reduced expression of the genera *Oxalobacter* found in UC may account for the development of hyperoxaluria and contribute to kidney stones in these patients.^7^

A large sample size is certainly a plus for the reliability of the data of the present study. Moreover, three statistical methods were used, and only results confirmed by all these three methods were included. This certainly made the results more robust and allowed to overcome the limitation of the different statistics used in single cohorts. The analysis was also adjusted for sequencing depth as the lowest threshold for inclusion was set.

This work has the merit of highlighting, on the one hand, some limitations related to microbiota analysis and, on the other hand, the potential of unifying and making data from different studies homogenous, which enabled the identification of new genera associated with IBD. The results of this work, along with a large portion of other papers on this topic, are limited by the lack of detailed clinical data that prevent subanalyses by disease type, location, activity, and concomitant therapies. The lack of information on diet and lifestyle habits is a further limitation. Certainly, this paper represents a starting point for future projects that, in addition to the possibility of improving the analysis with some clinical correlates, should be devoted to understanding the possible functional significance of the many novel associations identified in IBD patients.

## CONFLICT OF INTEREST STATEMENT

IM received fees from Janssen, Galapagos, Abbvie; GM served as a consultant for First Wave BioPharma, as a speaker for Takeda, Abbvie, Galapagos, and Pfizer, and filed a patent related to the treatment of inflammatory bowel diseases with Smad7 antisense oligonucleotides.

## Data Availability

Data sharing is not applicable to this article as no new data were created or analyzed in this study.

## References

[1] Teofani A , Marafini I , Laudisi F , Pietrucci D , Salvatori S , Unida V , et al. Intestinal taxa abundance and diversity in inflammatory bowel disease patients: an analysis including covariates and confounders. Nutrients. 2022;14(2):260. 10.3390/nu14020260 35057440 PMC8778135

[2] Halfvarson J , Brislawn CJ , Lamendella R , Vazquez‐Baeza Y , Walters WA , Bramer LM , et al. Dynamics of the human gut microbiome in inflammatory bowel disease. Nat Microbiol. 2017;2(5):17004. 10.1038/nmicrobiol.2017.4 28191884 PMC5319707

[3] Sinha R , Abnet CC , White O , Knight R , Huttenhower C . The microbiome quality control project: baseline study design and future directions. Genome Biol. 2015;16(1):276. 10.1186/s13059-015-0841-8 26653756 PMC4674991

[4] Sinha R , Abu‐Ali G , Vogtmann E , Fodor AA , Ren B , Amir A , et al. Assessment of variation in microbial community amplicon sequencing by the Microbiome Quality Control (MBQC) project consortium. Nat Biotechnol. 2017;35(11):1077–1086. 10.1038/nbt.3981 28967885 PMC5839636

[5] Vestergaard MV , Allin KH , Eriksen C , Zakerska‐Banaszak O , Arasaradnam RP , Alam MT , et al. Gut microbiota signatures in inflammatory bowel disease. United Eur Gastroenterol J. 2023. 10.1002/ueg2.12485 PMC10859715 38041519

[6] Mottawea W , Chiang CK , Muhlbauer M , Starr AE , Butcher J , Abujamel T , et al. Altered intestinal microbiota‐host mitochondria crosstalk in new onset Crohn's disease. Nat Commun. 2016;7(1):13419. 10.1038/ncomms13419 27876802 PMC5122959

[7] Kumar R , Ghoshal UC , Singh G , Mittal RD . Infrequency of colonization with *Oxalobacter formigenes* in inflammatory bowel disease: possible role in renal stone formation. J Gastroenterol Hepatol. 2004;19(12):1403–1409. 10.1111/j.1440-1746.2004.03510.x 15610315

